# Treatment of lymphatic leakage after retroperitoneal tumor resection by lymphangiography and embolization

**DOI:** 10.1016/j.ijscr.2019.12.030

**Published:** 2019-12-26

**Authors:** Gao Jiawei, Chen Wei

**Affiliations:** Department of General Surgery, Second Affiliated Hospital of Suzhou University, Suzhou 215004, PR China

**Keywords:** Lymphatic leakage, Retroperitoneal tumor, Treatment, Lymphangiography and embolization

## Abstract

Lymphatic leakage is an infrequent but relatively serious complication after resection of retroperitoneal tumors. Different approaches have been attempted in treatment of lymphatic leakage. However, to date none of them have been demonstrated consistently effective. We hereby report our preliminary experience with Lymphangiography and embolization for resolution of Lymphatic leakage after retroperitoneal tumor resection.

The patient, a 55-year-old woman with massive retroperitoneal tumor, Computed tomography (CT) revealed that retroperitoneal cystadenoma. After the right ureteral stent was placed, the retroperitoneal tumor resection was performed, postoperatively she presented with lymphatic leakage unresponsive to several treatment measures. From the thirty-seven postoperative day (POD37), the patient underwent Lymphangiography and embolization, which resolved her lymphatic leakage.

Lymphangiography is useful for detecting lymphatic leakage occurring after retroperitoneal tumor resection. Furthermore, lymphatic embolization is feasible, effective, and safe for managing leaks demonstrated on lymphangiography.

## Highlights

• This is a rate case of lymphatic leakage after the retroperitoneal giant tumor, which is rare in literature.
• A variety of methods adopted for non-surgical treatment, including the relatively new lymphangiodochography and embolization.
• Through the joint efforts of doctors and patients, patient was finally treated with conservative treatment and discharged.

## 1 Introduction

The work has been reported in line with the SCARE criteria [1].

The treatment of retroperitoneal tumor is frequently surgical resection and lymph node dissection. Lymphatic leakage following this is a common occurrence. A variety of approaches have been attempted to the treatment of lymphatic leakage. However, so far none has been consistently effective or optimal [2]. We hereby report a case of Lymphatic leakage after retroperitoneal tumor resection that was successfully resolved by Lymphangiography and embolization.

## 2 Case presentation

**Fig. 1:**
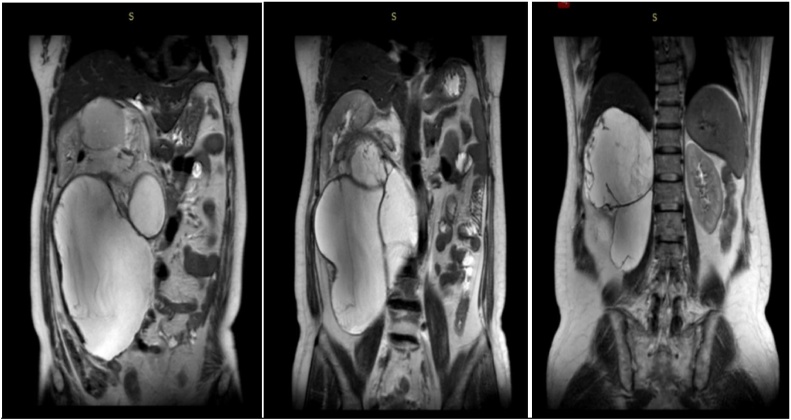
This coronal MRI of the abdomen shows a large retroperitoneal tumor. The organs have deviated from their normal position.

A 55-year-old female patient visited our hospital, who reported to have abdominal mass and complained that the mass gradually increased to affect sleep. B-ultrasound examination revealed mixed echo zone with approximately 243 × 118 in size in the abdominal cavity. Computed tomography (CT) showed a large mass on the right side of the abdomen, with the right ureter and inferior vena cava compressed to the right, indicating retroperitoneal cystadenoma. Magnetic Resonance Imaging (MRI) suggested a large multilocular cystic space in the retroperitoneal and hepatorenal space, approximately 261 × 181 × 150 mm in size, which was very likely to be epidermoid cyst (Fig. 1). After admission, physical examination showed a hard, local uplift in the right abdomen, about 25 × 12 cm in size, without tenderness, fixation or any other positive signs. After placing the right ureteral stent, the retroperitoneal tumor resection was performed. As a result, intraoperative exploration revealed a large cystic solid tumor in the right abdominal cavity, which was multilocular and lobulated. The inferior vena cava and ureter were pushed up to the right abdominal wall and the right kidney was moved up to the lower part of the liver. After cautious separation along the tumor to protect the blood vessels and ureters, careful irrigation was performed before the peritoneum was closed. And no active bleeding or obvious lymphatic leakage was detected. Pelvic cavity was then placed, followed by placing two drainage tubes at the incision.

**Fig. 2:**
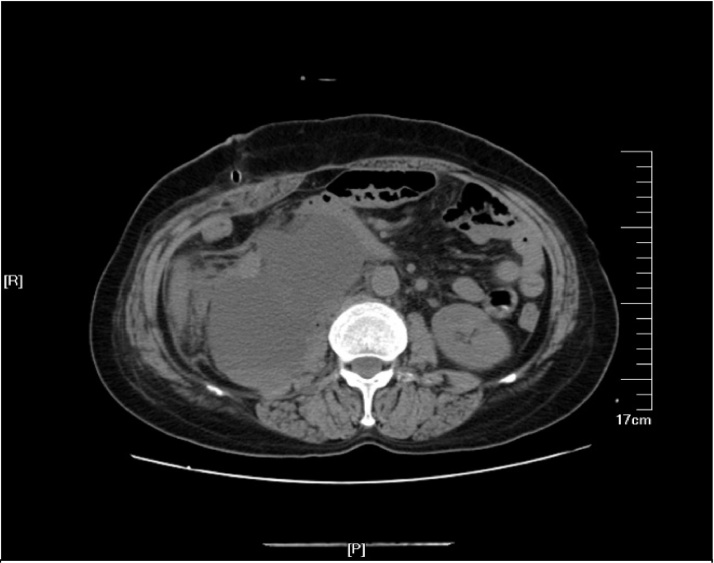
Abdominal CT scan shows a localized retroperitoneal Lymphatic leakage.

**Fig. 3:**
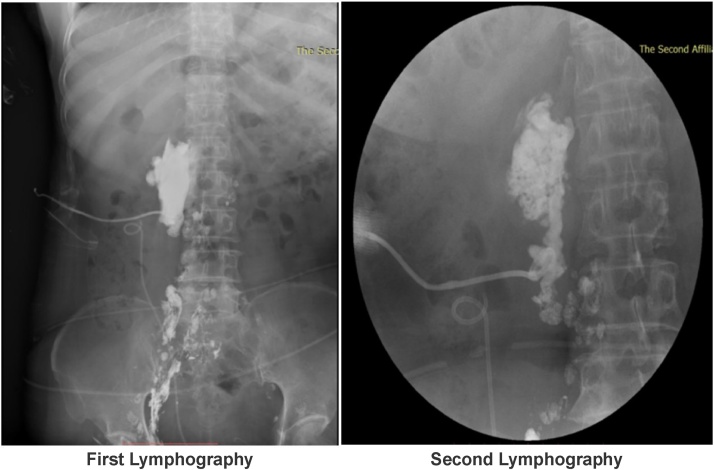
Lymphangiography showed massive exudation of lymph, the leakage was reduced on POD37 compared with POD30, but it was not completely occluded. A: First Lymphography. B: Second Lymphography.

Postoperative pathology :(posterior peritoneal) mature cystic teratoma showed mucinous tumor and mild to moderate atypical hyperplasia of glandular epithelium. After the operation, the drainage of the abdominal drainage tube was in light blood liquid in small amount. CT revealed peritoneal effusion after operation on POD7 (Fig. 2), therefore, peritoneocentesis was performed, which extracted 200 ml of yellow-white, turbidity liquid. Two abdominal drainage tubes were removed on the same day. After two days of observation, there was no decrease in the amount of abdominal drainage fluid. The chylous qualitative test of concurrent drainage fluid was performed, with positive Sudan staining. Therefore, the patient was instructed to eat high-calorie, high-protein, low-fat fluid, in order to strengthen parenteral nutrition, keep the drainage tube unobstructed. In addition, electrolyte was regularly reviewed to prevent water and electrolyte balance disorder, and the daily drainage volume was maintained between 700–1100 ml thereafter. Subsequently, in the first month after operation, lymph node lipiodolography and embolization were performed under ultrasound guidance. Daily drainage was decreased after lipiodolography, which was not obvious. One week later, the lymph node lipiodolography and embolization were re-performed (Fig. 3). As shown, the leakage of the exudation site was reduced. One day after the lipiodolography, 125 ml of milky liquid was drained, which suggested obviously covered leakage area and relatively limited scope.

Afterwards, the patient was instructed to fast and switch to total parenteral nutrition for one week. The drainage tube was clamped and the patient did not complain of any discomfort. After two weeks of lipiodolography, the peritoneal puncture drainage tube was removed, and the patient was instructed to eat a light diet and gradually make a transition. After one week of observation, no peritoneal effusion was found by b-ultrasound and the patient discharged from hospital. The patient was reviewed CT regularly after discharge, no abnormalities were found.

## 3 Discussion

Lymphatic leakage is a rare and well-known complication following retroperitoneal tumor resection. Great attempt has been made to the treatment of lymphatic leakage, as listed in the following. (1) Abdominal drainage. In spite of no direct evidence supporting that abdominal drainage can promote the healing of fistula, it can provide clinical diagnosis basis and alleviate a series of clinical symptoms such as abdominal pain and distension to a certain extent. In addition, the therapeutic treatment plan can be adjusted according to the amount of drainage fluid. (2) Antisecosis. Mid-chain triglyceride diet with parenteral nutrition can reduce the amount of lymphatic leakage, which is because the short-chain triacylglycerol contained in food can be absorbed directly into the blood through the intestinal tract, while the long-chain triacylglycerol should be transported and absorbed through the lymphatic pathway [3]. (3) Octreotide and somatostatin. It has been reported that the addition of octreotide in food exert a significantly early scavenging effect on postoperative lymphatic drainage of patients, mainly to prevent the conversion of triglycerides in the diet into free fatty acids in the intestinal tract, thereby reducing the absorption of fatty acids [4]. In recent years, surgical intervention has been reported to be guided by near-infrared fluorescence imaging technology, and indocyanine green can be used to locate leakage hot spots, providing high sensitivity and real-time imaging to help surgeons perform preventive ligation in cases where it is needed.This technique may have the potential to more accurately diagnose and treat lymphatic leakage during surgery [5].

**Fig. 4:**
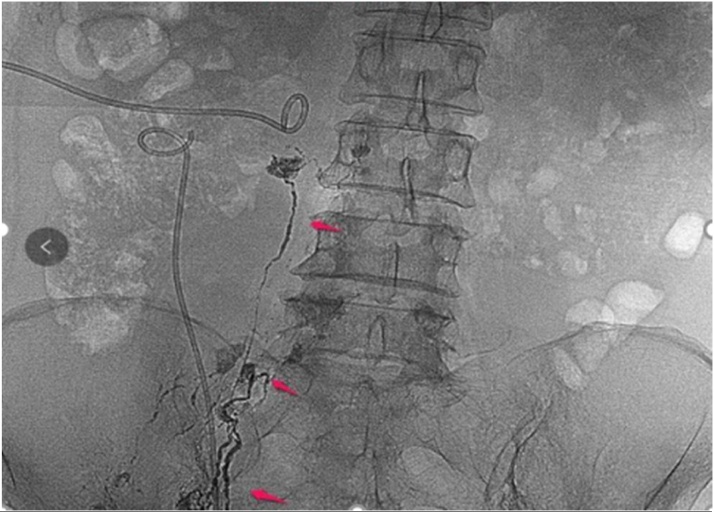
At the arrow we can see thin strips of lymphatic vessels and leakage development.

Nevertheless, some leaks still persist despite conservative treatment, therefore, more effective treatments are needed [6]. Lymphatic intervention is less invasive compared to surgery, involving injection of ethiodized oil into the lymphatic system to obtain a lymphangiogram [7]. In addition to its diagnostic value, lymphangiography has also been reported to have therapeutic effects [8]. This is possibly due to the high viscosity of contrast medium such as ethiodized oil, which can stimulate the growth of local new granulation tissue,triggering a series of inflammatory reactions to decrease leakage. In this case report, two procedures of lymphangiography and embolization were performed, in which thin strips of lymphatic vessels and leakage development were observed, however, without immediate effect (Fig. 4). The second attempt was successful and the amount of drainage was decreased from a maximum output of 700 to 125 ml/day after lymphangiography.

Our case demonstrates that postoperative lymphatic leakage that was successfully treated by performing repeated lymphangiography and embolization. This technique should be considered for further use and investigation for abdominal surgery and lymph node dissections.

## Availability of data and materials

The datasets used and/or analyzed during the current study are available from the corresponding author on reasonable request.

## Declaration of Competing Interest

The authors declare that they have no competing interests.

## Funding

No funding was received.

## Ethical approval

The study was approved by the Ethics Committee of Second Affiliated Hospital of Suzhou University, China. Patients who participated in this research, signed the informed consent and had complete clinical data. Signed written informed consents were obtained from the patients and/or guardians.

## Consent

Written informed consent was obtained from the patient for publication of this case report and accompanying images. A copy of the written consent is available for review by the Editor-in-Chief of this journal on request.

## Author contribution

JG wrote the manuscript, interpreted and analyzed the data. WC designed the study and performed the experiment. JC was responsible for the analysis and discussion of the data. Both authors read and approved the final manuscript.

## Guarantor

Dr. Chen Wei.

## Provenance and peer review

Not commissioned, externally peer-reviewed.
